# Micro-Structures and High-Temperature Friction-Wear Performances of Laser Cladded Cr–Ni Coatings

**DOI:** 10.3390/ma11010137

**Published:** 2018-01-15

**Authors:** Li Jiahong, Kong Dejun

**Affiliations:** 1 College of Mechanical Engineering, Changzhou University, Changzhou 213164, China; lijiahonglyh@163.com; 2 Changzhou High Technology Research Key Laboratory of Mould Advanced Manufacturing, Changzhou University, Changzhou 213164, China

**Keywords:** Cr–Ni coating, laser cladding (LC), coefficient of friction (COF), high temperature wear, wear mechanism

## Abstract

Cr–Ni coatings with the mass ratios of 17% Cr–83% Ni, 20% Cr–80% Ni and 24% Cr–76% Ni were fabricated on H13 hot work mould steel using a laser cladding (LC). The surface–interface morphologies, chemical elements, surface roughness and phase composition of the obtained Cr–Ni coatings were analysed using a scanning electron microscope (SEM), energy disperse spectroscopy (EDS), atomic force microscope (AFM) and X–ray diffractometer (XRD), respectively. The friction–wear properties and wear rates of Cr–Ni coatings with the different mass ratios of Cr and Ni at 600 °C were investigated, and the worn morphologies and wear mechanism of Cr–Ni coatings were analysed. The results show that the phases of Cr–Ni coatings with mass ratios of 17% Cr–83% Ni, 20% Cr–80% Ni and 24% Cr–76% Ni are composed of Cr + Ni single-phases and their compounds at the different stoichiometry, the porosities on the Cr–Ni coatings increase with the Cr content increasing. The average coefficient of friction (COF) of 17% Cr–83% Ni, 20% Cr–80% Ni and 24% Cr–76% coatings are 1.10, 0.33 and 0.87, respectively, in which the average COF of 20% Cr–80% Ni coating is the lowest, exhibiting the better anti-friction performance. The wear rate of 17% Cr–83% Ni, 20% Cr–80% Ni and 24% Cr–76% Ni coatings is 4.533 × 10^−6^, 5.433 × 10^−6^, and 1.761 × 10^−6^ N^−1^·s^−1^, respectively, showing the wear resistance of Cr–Ni coatings at a high temperature increases with the Cr content, in which the wear rate is 24% Cr–76% Ni coating with the better reducing wear. The wear mechanism of 17% Cr–83% Ni and 20% Cr–80% Ni and 24% Cr–76% coatings at 600 °C is primarily adhesive wear, and that of 24% Cr–76% coating is also accompanied by oxidative wear.

## 1. Introduction

As a hot work mould steel, H13 steel (4Cr5MoSiV1) with high wear resistance, thermal stability and fatigue resistance, etc. [1] is widely used on hot forging, hot extrusion and hot casting mould manufacturing [2], of which comprehensive mechanical properties are well at medium temperature of ~550 °C [3]. The microstructure of H13 steel at high temperature of ≥550 °C is changed constantly; its hardness declines rapidly, which affects its wear properties [2,4]. At the same time, the mould surface will have wear failure, thermal fatigue cracking and other failures [1], due to strong friction and mechanical impact at high temperature [2]. Thermal spraying with high efficiency, low cost and easy controlling is often used to improve the mould wear resistance [5,6], but it has some defects such as low material utilization, large waste and low bonding strength, etc. As an advanced surface technology, the laser cladded coatings are metallurgically bonded with the substrate after alloy powder rapid melting and solidification [2], which is suitable for a variety of moulds. The LC has several outstanding advantages in the surface modification field such as refined microstructures, metallurgical bonding and narrow heat affected zone (HAZ) [7,8], etc. Laser cladding improves the bonding strength between the coating and the substrate comparing to thermal spraying and reduces the loss of the coating during wear [9]. Coatings can greatly improve the wear resistance of the surface of the material to improve its wear properties [10,11], particularly compared to the substrate [12]. The composition of Ni and Cr affects the wear resistance of Cr–Ni coating [13,14]; research on the Cr–Ni coating is mainly concentrated on its anti-oxidation. Bala et al. [15] investigated the oxidation behaviour of Ni–20Cr and Ni–50Cr coatings in the cycle of heating and cooling conditions. Zhang et al. [16] analysed the oxidation resistance of Ni–Cr nano-composite films at high temperature. The above Cr–Ni coatings were fabricated using an electro-deposition; there were few reports on the friction-wear properties of laser cladded Cr–Ni coatings with the different mass ratios of Cr and Ni at high temperature. In this study, the Ni–Cr coatings with the mass ratios of 17% Cr–83% Ni, 20% Cr–80% Ni and 24% Cr–76% Ni were fabricated using an LC on H13 hot work mould steel. The morphologies, chemical elements, phases and surface roughness of the obtained Cr–Ni coatings were analysed using a scanning electronic microscopy (SEM), energy disperse spectroscopy (EDS), X–ray diffractometer (XRD) and atomic force microscopy (AFM), respectively. The effects of mass ratios of Cr and Ni on high temperature friction-wear performances of laser-cladded Cr–Ni coating were discussed, and the mechanism of Cr–Ni coating also analysed, which provided an experimental basis for the surface modification of H13 hot work mould steel.

## 2. Experiment

The substrate was H13 hot work mould steel; its chemical composition is shown in Table 1. Three kinds of powders were differentiated by mass ratios of Cr and Ni as follows: 17% Cr–83% Ni, 20% Cr–80% Ni and 24% Cr–76% Ni. The morphologies and chemical compositions of Cr–Ni powders were analysed using an JSM-6360LA type SEM (NEC Electronics Corporation, Tokyo, Japan) and its configured EDS, respectively, and the phase compositions of Cr–Ni powders were analysed using a D/max 2500PC type XRD (Rigaku Corporation, Tokyo, Japan).

The above three kinds of Cr–Ni powders were respectively covered on H13 steel with the thickness of 2 mm, the acetone solution was dropped on the surfaces of Cr–Ni powders using a dropper. After drying at room temperature for 5 h, the LC test was carried out on a ZKSX-2008 type fiber-coupled of laser spraying system (Jiangsu Zhongke Sixiang Laser Technology Co., Ltd., Danyang, China), the technological parameters were: focal length of laser beam is 400 mm, laser power of 1400 W, spot diameter of 4 mm, moving speed of 10 m/s, Argon was used as the shielding gas during the LC test. After the LC test, the surface–interface morphologies and chemical compositions of the obtained Cr–Ni coatings were analysed using a JSM-6360LA type SEM and its configured EDS, the surface roughness was analysed using a CSPM5500 type AFM (Original Nanoscale Instrument Co., Ltd., Beijing, China), and the phase compositions of Cr–Ni coatings were analysed using a D/max 2500PC type XRD. The microhardness of the coatings was measured using a HMV-2T type Vickers microhardness tester (SHIMADZU enterprise management (China) Co., Ltd., Shanghai, China) with the load of 2N and the loading time of 10 s. The friction and wear test were conducted on a HT-1000 type high temperature friction-wear tester (Lanzhou Zhongke Kaihua Science and Technology Development Co., Ltd., Lanzhou, China) at 600 °C. The wear test parameters: friction method of sliding friction, motor frequency of 8.93 Hz, friction-pair of ceramic ball with the diameter of 3 mm load of 5 N, speed of 500 rpm, rotation radius of 4 mm, duration time of 30 min. After the wear test, the profiles of worn tracks were analysed using a VHX-700FC type super–depth three-dimensional microscopic system (Keyence Corporation, Osaka, Japan), and the morphologies and energy spectrum of worn tracks were analysed using a SEM and its configured d EDS, respectively.

## 3. Analysis and Discussion

### 3.1. Morphologies, EDS and XRD Analysis of Cr–Ni Powder

**Figure 1:**
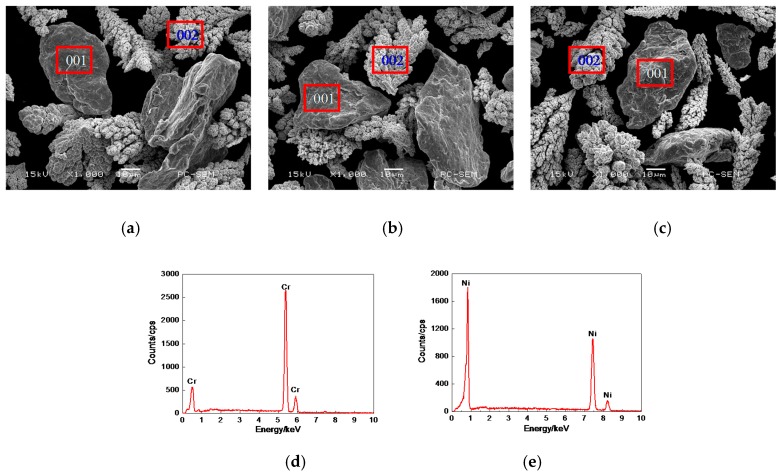
Morphologies and EDS analysis of Cr–Ni powders with different mass ratios of Cr and Ni. (**a**) Morphology of 17% Cr–83% Ni powder; (**b**) Morphology of 20% Cr–80% Ni powder; (**c**) Morphology of 24% Cr–76% Ni powder; (**d**) EDS analysis at 001 point; (**e**) EDS analysis at 002point.

The morphologies of 17% Cr–83% Ni and 20% Cr–80% Ni and 24% Cr–76% Ni powders are shown in Figure 1a–c. Their shapes were basically similar, indicating that the mass ratios of Cr and Ni had no obvious effects on the powder morphologies. The point of 001 was Cr powder, whose surface was smooth and had lines, the result of EDS analysis is shown in Figure 1d. The point of 002 was Ni powder, whose surface was pine-cone-shaped, the result of EDS analysis is shown in Figure 1e. From the above analyses, it can be seen that the Cr–Ni powder was composed of pure Cr and Ni powders, without other impurities.

**Figure 2:**
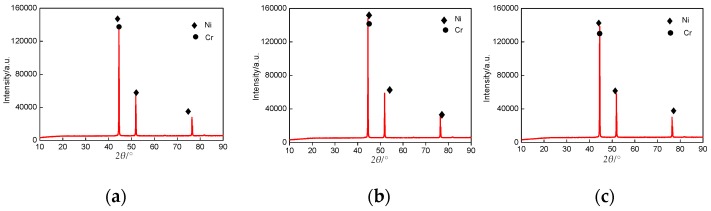
XRD patterns of Cr–Ni powders with different mass ratios of Cr and Ni. (**a**) XRD patterns of 17% Cr–83% Ni powder; (**b**) XRD patterns of 20% Cr–80% Ni powder; (**c**) XRD patterns of 24% Cr–76% Ni powder.

The XRD patterns of Cr–Ni powders with different mass ratios of Cr and Ni are shown in Figure 2a–c. There were no obvious differences at the XRD diffraction peaks, indicating that the Cr–Ni powders with different ratios of Cr and Ni were all composed of Cr and Ni phases.

### 3.2. Morphologies of Cr–Ni Coating Surface

**Figure 3:**
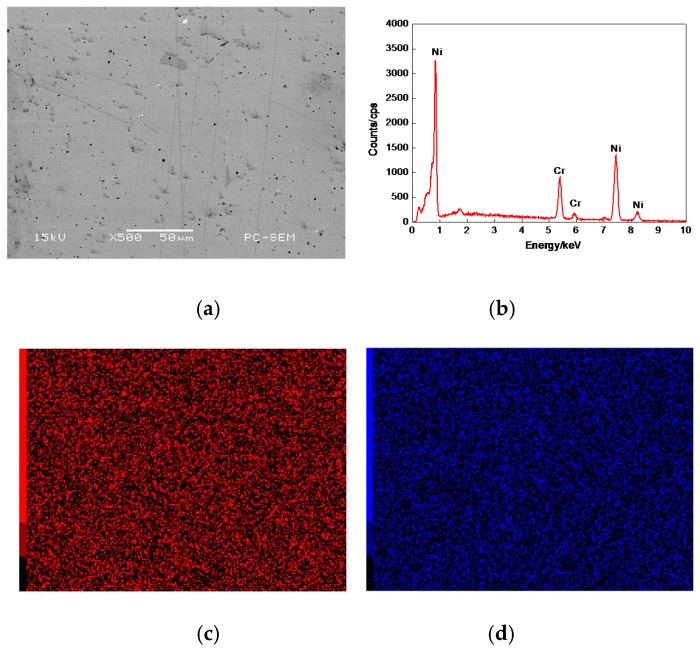
Plane scan analysis of 17% Cr–83% Ni coating surface. (**a**) Plane scanned position; (**b**) Plane scan analysis 17% Cr–83% Ni coating; (**c**) Distribution of Cr content; (**d**) Distribution of Ni content.

Figure 3a shows the morphology of 17% Cr–83% Ni coating surface, which was relatively smooth with fine porosities and no obvious defects. The plane scan analysis result of 17% Cr–83% Ni coating is shown in Figure 3b, indicating that the 17% Cr–83% Ni coating was composed of Cr and Ni, with no other elements. The Cr and Ni were uniformly distributed, as shown in Figure 3c,d.

**Figure 4:**
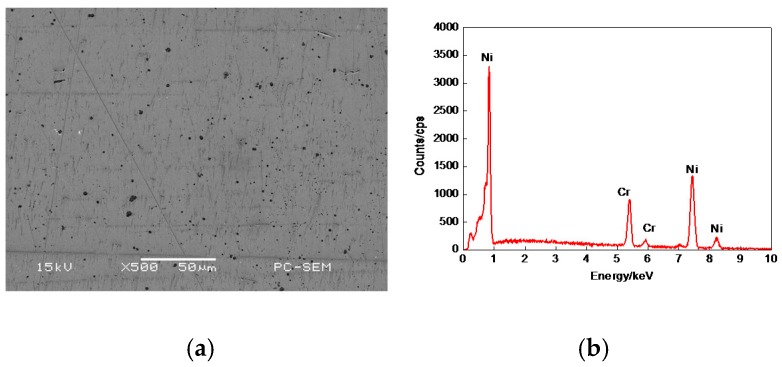

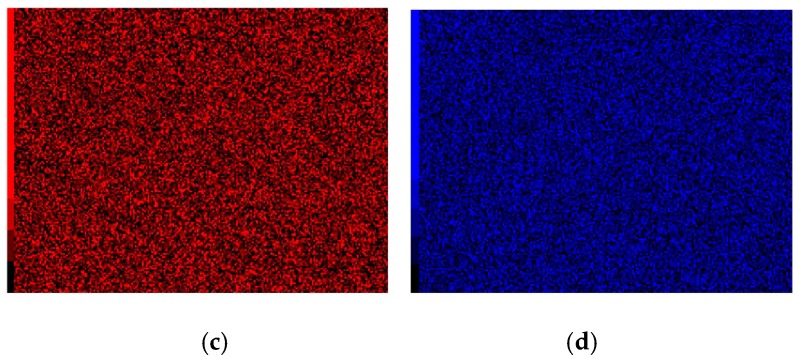
Plane scan analysis of 20% Cr–80% Ni coating surface. (**a**) Plane scanned position; (**b**) Plane scan analysis 20% Cr–80% Ni coating; (**c**) Distribution of Cr content; (**d**) Distribution of Ni content.

The morphology of 20% Cr–80% Ni coating surface is shown in Figure 4a, which was similar to that in Figure 3a, but a few porosities were larger. The plane scan analysis result of 20% Cr–80% Ni coating is shown in Figure 4b, indicating that the 20% Cr–80% Ni coating was composed of Cr and Ni, with no other elements. The distributions of Cr and Ni are shown in Figure 4c,d, with no obvious atom-poor zones.

**Figure 5:**
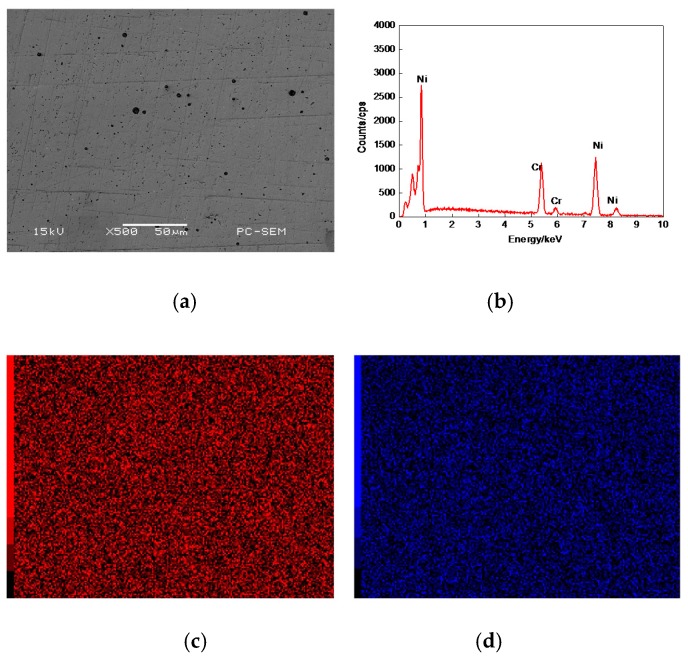
Plane scan analysis of 24% Cr–76% Ni coating surface. (**a**) Plane scanned position; (**b**) Plane scan analysis 24% Cr–76% Ni coating; (**c**) Distribution of Cr content; (**d**) Distribution of Ni content.

The morphology of 24% Cr–76% Ni coating surface is shown in Figure 5a. Compared with Figure 3a and Figure 4a, the porosity sizes became larger. The plane scan analysis result of 24% Cr–76% Ni coating is shown in Figure 5b, indicating that the 24% Cr–76% Ni coating was also composed of Cr and Ni, with no other impurity elements. The distributions of Cr and Ni are shown in Figure 5c,d, which are the same as those of 17% Cr–83% Ni and 20% Cr–80% Ni coatings, with no evident difference. Comparing the morphologies of surfaces in Figure 3, Figure 4 and Figure 5a, the porosities became larger, which were related to the Cr content. The forming of porosities was because of the presence of an eddy flux, forward and downward, around the key hole interface; the formed bubbles do not escape from the molten pool, but remain entrapped, thus generating porosity at the end of the solidification process [17,18,19], and another reason for porosity generation was that the free carbons in the powders were easily combined with the O in the air to form CO or CO_2_ [19].

### 3.3. Line Scan Analysis of Cr–Ni Coating Surface

**Figure 6:**
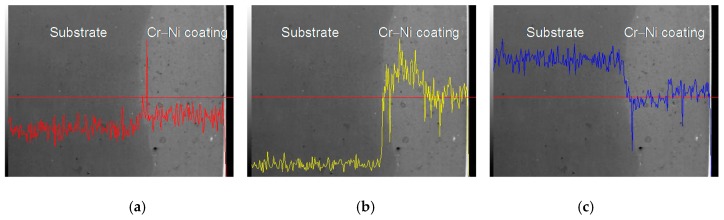
Line scan analysis of 17% Cr–83% Ni coating interface. (**a**) Line scan analysis of Cr content; (**b**) Line scan analysis of Ni content; (**c**) Line scan analysis of Fe content.

**Figure 7:**
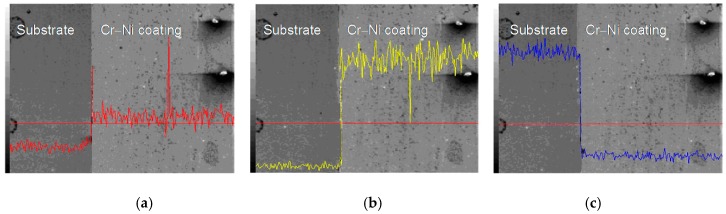
Line scan analysis of 20% Cr–80% Ni coating interface. (**a**) Line scan analysis of Cr content; (**b**) Line scan analysis of Ni content; (**c**) Line scan analysis of Fe content.

**Figure 8:**
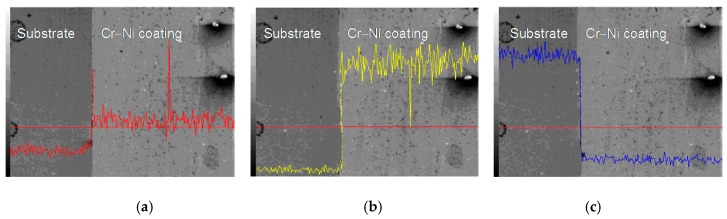
Line scan analysis of 24% Cr–76% Ni coating interface. (**a**) Line scan analysis of Cr content; (**b**) Line scan analysis of Ni content; (**c**) Line scan analysis of Fe content.

The line scan analyses of 17% Cr–83%, 20% Cr–80% Ni and 24% Cr–76% Ni coating interfaces are shown in Figure 6, Figure 7 and Figure 8. The Cr content of 17% Cr–83% Ni coating was higher than that of the substrate, as shown in Figure 6a. The Ni of 17% Cr–83% Ni coating maintained high content, while that of substrate maintained low content; the metallurgical bonding layer was formed at the coating–substrate interface, as shown in Figure 6b. The Fe content was high in the substrate, and declined at the diffusion layer, as shown in Figure 6c.

The line scan analysis of 20% Cr–80% Ni coating interface is shown in Figure 7. The Cr content of 20% Cr–80% Ni coating increased slightly compared with that of the substrate, as shown in Figure 7a. The Ni maintained low content in the substrate, and increased to a high value at the metallurgical bonding layer of 20% Cr–80% Ni coating, as shown in Figure 7b. The Fe content of the substrate was far higher than that of 20% Cr–80% Ni coating, and dropped to a small value at the diffusion layer, as shown in Figure 7c.

The line scan analysis of 24% Cr–76% Ni coating interface is shown in Figure 8. The Cr contents of 24% Cr–76% Ni coating and the substrate changed significantly, which decreased rapidly at the diffusion layer, as shown in Figure 8a. The Ni maintained a lower content in the substrate, and increased to a high value at the metallurgical bonding layer of 20% Cr–80% Ni coating, as shown in Figure 8b. On the contrary, Fe had a higher content in the substrate and decreased at the diffusion layer, as shown in Figure 8c.

From the above line scan analyses, it can be known that the Cr and Ni formed the atom-rich zones in the three kinds of Cr–Ni coatings and the diffusion layer at the coating–substrate interface, indicating that the metallurgical bonding was produced between the coating and the substrate after LC.

### 3.4. AFM Analysis of Cr–Ni Coating Surface

**Figure 9:**
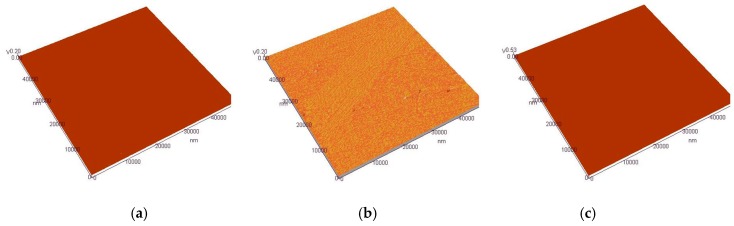
AFM topographies of Cr–Ni coatings with different mass ratios of Cr and Ni.(**a**) AFM topography of 17% Cr–83% Ni coating; (**b**) AFM topography of 20% Cr–80% Ni coating; (**c**) AFM topography of 24% Cr–76% Ni coating.

The topographies of three kinds of Cr–Ni coatings were measured using an AFM, as shown in Figure 9a–c. The image size was 50,000 nm × 50,000 nm. The parameters such as contour arithmetic mean deviation of *S*_a_, surface morphology root mean square deviation of *S*_q_, surface height distribution inclination *S*_sk_, surface height distribution kurtosis *S*_ku_ and surface ten-point height *S*_z_ were used to characterize the three-dimensional micro-topography; their values are shown in Table 2. There were no significant differences in the surface roughness of Cr–Ni coatings; the effect on the friction-wear properties was negligible due to their small magnitude grade.

### 3.5. XRD Analysis of Cr–Ni Coatings

**Figure 10:**
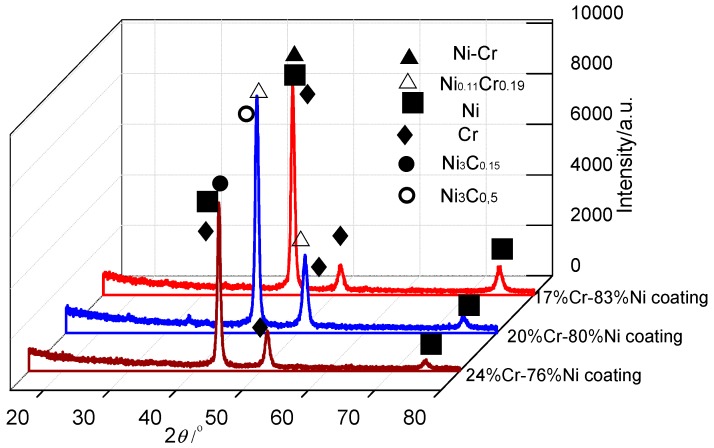
XRD patterns of Cr–Ni coatings with different mass ratios of Cr and Ni.

The XRD patterns of Cr–Ni coatings with different mass ratios are shown in Figure 10. The phases of 17% Cr–83%, 20% Cr–80% Ni and 24% Cr–76% Ni coating were composed of Ni–Cr + Ni + Cr, Ni_0.11_Cr_0.19_ + Ni_3_C_0.5_ + Ni + Cr, and Ni_3_C_0.15_ + Ni + Cr, respectively, indicating that there are Cr + Ni single-phases and their compounds in the Ni Cr–Ni coatings, which were at a different stoichiometry. This was because there were different mass ratios of Cr and Ni in the Cr–Ni coatings; the Ni was not only reacted with the Cr to form the above compounds of Ni–Cr and Ni_0.11_Cr_0.19_, but also easily reacted with the C in the air to form the Ni carbides of Ni_3_C_0.5_ and Ni_3_C_0.15_.

### 3.6. Friction and Wear Performances

#### 3.6.1. Coefficients of Friction

**Figure 11:**
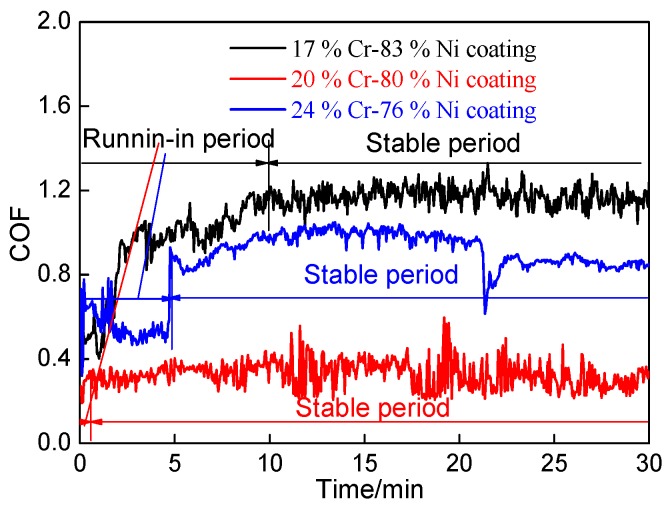
COFs of Cr–Ni coatings vs wear time.

The relationships between the coefficients of friction (COFs) of Cr–Ni coatings with different ratios of Cr and Ni and the wear time are shown in Figure 11. The average COF of 17% Cr–83% Ni, 20% Cr–80% Ni and 24% Cr–76% Ni coating was 1.10, 0.33, and 0.87, respectively. The wear process was divided into running-in period and stable wear period, there was no wear failure period. The average COF of 17% Cr–83% Ni coating was 0.93 in the running-in period (0–10 min), which increased rapidly from 0.50 to 1.03 in the time range of 0–3 min and then slowly increased to 1.19 at 10 min. The COF of 20% Cr–80% Ni coating increased from 0.27 to 0.31 in the running–in period (0–0.5 min), the average COF was 0.28. When entering into the stable wear period (0.5–30 min) with no obvious fluctuation, the average COF was 0.33. The average COF of 24% Cr–76% Ni coating increased rapidly from 0.32 to 0.87 in the running–in period (0–5 min), which were obviously fluctuating, and its average COF was 0.33. The average COF was 0.92 in the stable wear period (5–30 min); in the first stable wear period (5–21.25 min), the average COF was 0.94, and then the COF was reduced from 0.91 to 0.75 at 21.25–21.30 min. In the second stable wear period (21.3–30 min), the average COF dropped to 0.85. From the above analyses, it can be seen that the 20% Cr–80% Ni coating had a lower COF, which was three-times lower than 17% Cr–83% Ni and 24% Cr–76% Ni coatings. In addition, the plastic deformation at the concavo-convex joint was large, which led to higher COF and instability [20]. In addition, for the Cr–Ni coatings the oxidation reaction occurred at 600 °C, resulting in the oxides of NiO and Cr_2_O_3_ [21,22], i.e.,

2Ni + O_2_ = 2NiO (1)

4Cr + 3O_2_ = 2Cr_2_O_3_

When the above oxides were formed, the debris was generated on the worn track, causing the COFs of Cr–Ni coatings to oscillate [23]. Under the same oxidation conditions, the Cr_2_O_3_ in Equation (2) was formed prior to the NiO in Equation (1), this was because the Gibbs free energy of Cr_2_O_3_ was lower than that of NiO [24]. Therefore, the oxide debris of 24% Cr–76% Ni coating was the most, and it had the most obvious fluctuation.

#### 3.6.2. Profiles of Worn Tracks

**Figure 12:**
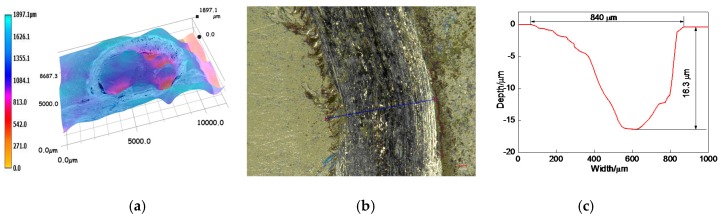
Profile of worn track on 17% Cr–83% Ni coating. (**a**) Overall profile of worn track; (**b**) Measured position of worn track; (**c**) Profile of worn track.

The overall profile of worn track on the 17% Cr–83% Ni coating is shown in Figure 12a. The measured position of worn track is shown in Figure 12b, and the profile of worn track is shown in Figure 12c. The wear width and wear depth were 840 and 16.3 μm, respectively. After calculation, the wear area and wear volume were 6.493 × 10^−3^ mm^2^, and 40.797 × 10^−3^ mm^3^, respectively, and the corresponding wear rate was 4.533 × 10^−6^ mm^3^·N^−1^·s^−1^.

**Figure 13:**
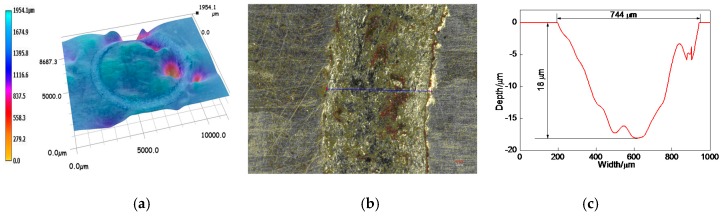
Profile of worn track on 20% Cr–80% Ni coating. (**a**) Overall profile of worn track; (**b**) Measured position of worn track; (**c**) Profile of worn track.

The overall profile of worn track on the 20% Cr–80% Ni coating is shown in Figure 13a. The measured position of worn track is shown in Figure 13b, and the profile of worn track is shown in Figure 13c. The wear width and wear depth of worn track were 744 and 18 μm, respectively. After calculation, the wear area and wear volume were 7.783 × 10^−3^ mm^2^ and 48.902 × 10^−3^ mm^3^, respectively, and the corresponding wear rate was 5.433 × 10^−6^ mm^3^·N^−1^·s^−1^.

**Figure 14:**
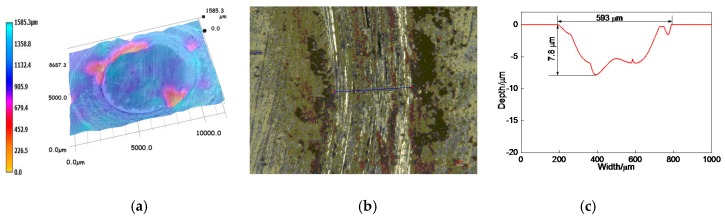
Profile of worn track on 24% Cr–76% Ni coating. (**a**) Overall profile of worn track; (**b**) Measured position of worn track; (**c**) Profile of worn track.

The overall profile of worn track on the 24% Cr−76% Ni coating is shown in Figure 14a. Figure 14b shows the measured position of worn track, and the profile of worn track is shown in Figure 14c. The wear width and wear depth were 593 and 7.8 μm, respectively. After calculation, the wear area and wear volume were 2.523 × 10^−3^ mm^2^ and 15.852 × 10^–3^ mm^3^, respectively, and the corresponding wear rate was 1.761 × 10^−6^ mm^3^·N^−1^·s^−1^.

According to the above analyses, the wear volumes of 17% Cr–83% Ni and 20% Cr–80% Ni coatings had no big difference, whereas the wear rate of 24% Cr–76% Ni coating was 2–3 times lower than those of 17% Cr–83% Ni and 20% Cr–80% Ni coatings, indicating that the wear performance of 17% Cr–83% Ni coating was the best.

#### 3.6.3. Worn Morphologies of Friction–pairs

**Figure 15:**
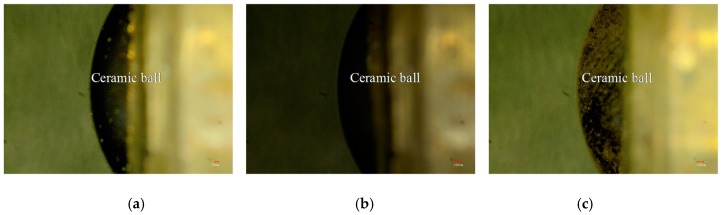
Worn morphologies of friction-pairs on Cr–Ni coatings with different mass ratios of Cr and Ni. (**a**) Worn morphology of friction-pair on 17% Cr–83% Ni coating; (**b**) Worn morphology of friction-pair on 20% Cr–80% Ni coating; (**c**) Worn morphology of friction-pair on 24% Cr–76% Ni coating.

Figure 15a–c shows the worn morphologies of friction-pairs on the three kinds of Cr–Ni coatings at 600 °C. The microhardness values of 17% Cr–83% Ni, 20% Cr–80% Ni, 24% Cr–76% Ni coatings were 369.6, 280.3 and 355.7 HV, respectively, while the microhardness of ceramic ball was 2200 HV, which was obviously higher than the coatings; therefore, the wear mainly occurred in the Cr–Ni coatings, rather than in the ceramic ball. The ceramic balls of friction pairs were not obviously worn; this was because the oxidation temperature of ceramic balls was 1300–1400 °C, while the experimental temperature was 600 °C, which decreased the micro–hardness of Cr–Ni coatings [21], and had no effect on the micro-hardness of ceramic ball.

### 3.7. Wear Mechanism

#### 3.7.1. Plane Scan Analysis of Worn Tracks

**Figure 16:**
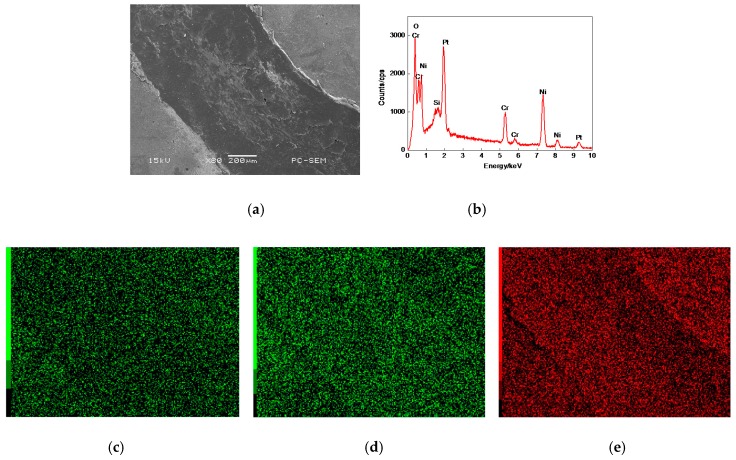
Plane scan analysis of worn track on 17% Cr–83% Ni coating. (**a**) Plane scanned position; (**b**) Plane scan analysis of worn track on 17% Cr–83% Ni coating; (**c**) Distribution of Cr content; (**d**) Distribution of Ni content; (**e**) Distribution of O content.

The plane-scanned position of worn track on the 17% Cr–83% Ni coating is shown in Figure 16a, which had a litter debris, obvious cracks and flake falling off. Figure 16b shows the mass fractions (mass, %) of plane scan analysis: Cr 8.86, Ni 86.15, O 5.00. The Pt and Si peaks appeared in the EDS result, among them, the Pt was the sprayed metal on the Cr–Ni coating surface to improve its conduction during the SEM test, and the Si came from the ceramic ball stuck on the worn track. The Cr and Ni were the elements of Cr–Ni coating, which were uniformly distributed on the worn track, as shown in Figure 16c,d. The O content accounted for 5.00% of gross mass fractions, which was the oxidation result of Cr–Ni coating at 600 °C; its distribution is shown in Figure 16e.

**Figure 17:**
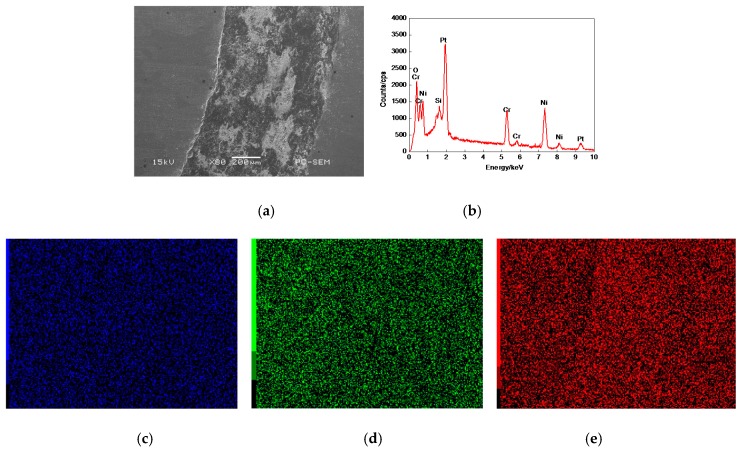
Plane scan analysis of worn track on 20% Cr–80% Ni coating. (**a**) Plane scanned position; (**b**) Plane scan analysis of worn track on 20% Cr–80% Ni coating; (**c**) Distribution of Cr content; (**d**) Distribution of Ni content; (**e**) Distribution of O content.

The plane scanned position of worn track on the 20% Cr–80% Ni coating is shown in Figure 17a, in which some debris was accumulated. Figure 17b shows the mass fractions (mass, %) of plane scan analysis: Cr 12.58, Ni 82.95, Si 1.06, and O 3.74. The Pt and Si also appeared on the worn track, whose origins were the same as that of 17% Cr–83% Ni coating. The Cr and Ni accounted for 95.58% of gross mass fractions, which were the primary compositions of Cr–Ni coating, their distributions were uniform with no atoms-rich zones, as shown in Figure 17c,d. The O accounted for 3.74% of gross mass fractions, which was uniformly distributed on the worn track with no atom-rich zones, as shown in Figure 17e.

**Figure 18:**
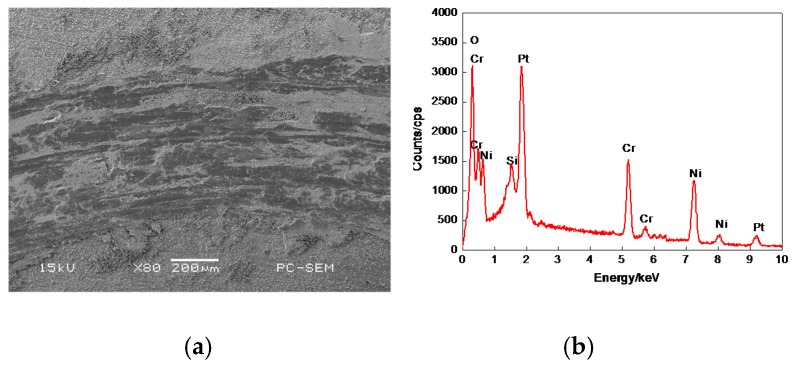

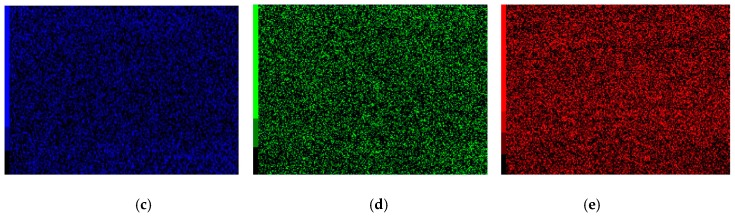
Plane scan analysis of worn track on 24% Cr–76% Ni coating. (**a**) Plane scanned position; (**b**) Plane scan analysis of worn track on 24% Cr–76% Ni coating; (**c**) Distribution of Cr content; (**d**) Distribution of Ni content; (**e**) Distribution of O content.

The plane scanned position of the worn track the of the 24% Cr–76% Ni coating is shown in Figure 18a, in which much debris was accumulated. Figure 18 (b) shows the mass fractions (mass, %) of plane scan analysis of Cr 13.32, Ni 81.74, Si 1.02, and O 4.84. The Pt was the result of sprayed metal during the SEM test, while the Si was the Si of ceramic ball sticking on the worn track. The Cr and Ni were also uniformly distributed on the worn track, as shown in Figure 18c,d. The O accounted for 4.845% of gross mass fractions, whose distribution is shown in Figure 18e.

Compared with the EDS results in Figure 16b, Figure 17b and Figure 18b and Figure 3a, Figure 4b and Figure 5b, the Cr–Ni coatings underwent an oxidation reaction at 600 °C, resulting in the oxides of NiO and Cr_2_O_3_ [21,22], i.e., most of the debris which was generated during wear remained in the form of loose particles, forming stick-slip friction [23]. When the Ni was alloyed with the Cr at sufficiently high concentrations, the Cr was selectively oxidized and the oxidation resistance increased by the Cr_2_O_3_ oxide below 800 °C [22]. The oxidation of Cr in the Cr–Ni coating was depended on the concentration of Cr and the diffusion rate. When the mass fraction of Cr was greater than 20% [22], the diffusion rate increased with the Cr content increasing. The mass fractions of Cr in the 20% Cr–80% Ni and 24% Cr–76% Ni coatings were greater than 20%; therefore, their oxidation resistance was better than the 17% Cr–83% Ni coating.

#### 3.7.2. Worn Morphologies

**Figure 19:**
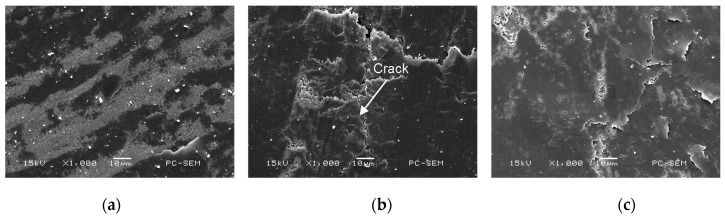
Morphologies of worn track on 17% Cr–83% Ni coating. (**a**) Adhesive wear; (**b**) Crack; (**c**) Spalling off.

In a case of the 17% Cr–83% Ni coating, the wear mechanism was adhesive wear, as shown in Figure 19a. There were different scratches and some broken debris on the worn track. The long and deep cracks were clear, as shown in Figure 19b. Although the debris was less in the wear test, the Cr–Ni coating had a spalling trend, as shown in Figure 19c.

**Figure 20:**
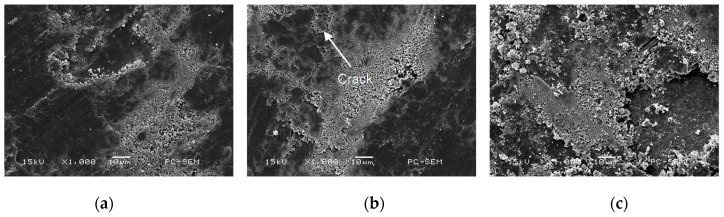
Morphologies of worn track on 20% Cr–80% Ni coating. (**a**) Adhesive wear; (**b**) Crack; (**c**) Spalling off.

There were some furrows on the 20% Cr–80% Ni coating; the wear mechanism was adhesive wear as shown in Figure 20a. The fine lattice-like cracks were formed on the worn track, as shown in Figure 20b. Compared with those in Figure 19b, the cracks were much finer and lighter. The large particles were spalled and accumulated, as shown in Figure 20c.

**Figure 21:**
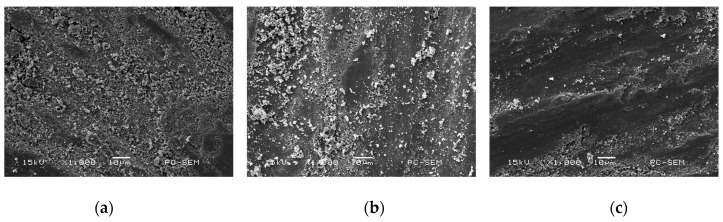
Morphologies of worn track on 24% Cr–76% Ni coating. (**a**) Adhesive wear; (**b**) Crack; (**c**) Spalling off.

The wear mechanism of 24% Cr–76% Ni coating was oxidative wear and adhesive wear, as shown in Figure 21a. The smooth regions (tribo-oxide layer) and the delaminated regions were found on the worn track, which was the typical characteristic of oxidative wear [25], as shown in Figure 21b. There was a large amount of deposits and large debris particles, which were mainly from the peeling of oxides when they were worn down [23]. Although the debris was much, no cracks were generated, as shown in Figure 21c, indicating that the 24% Cr–76% Ni coating was not obviously damaged.

When the friction pair was contacted with the Cr–Ni coating, the wear first occurred on a few independent asperities. Under the wear load, the local pressure of the asperity exceeded the yield pressure of Cr–Ni coating; plastic deformation occurred, so that the adhesion phenomenon appeared on the Cr–Ni coating surface. The relative movement led to cutting and breaking, the shedding of Cr–Ni coating became debris, and the adhesive-shear-transfer-re-adhesion phenomenon was continuously carried out to form adhesive wear, which was the wear process of 17% Cr–83% Ni coating. The wear test results showed that abrasive wear was apparent at room temperature, whereas adhesive wear was dominant at elevated temperatures [26].

The 24% Cr–76% Ni coating first experienced oxidized wear, with a great number of oxide particles appearing on the worn track, which at times were removed and at other times were compacted again, forming adhesion wear. The micro-hardness of Cr–Ni coatings decreased at high temperature [27], the fragile oxide film was prone to adhesive wear and debris falling off, in which the wear process existed on the 20% Cr–80% Ni coating. The Cr–Ni coating was oxidized into the fine-grained Cr_2_O_3_ at 600 °C, reducing the presence of cracks [22]. Therefore, no cracks appeared on the 24% Cr–76% Ni coating with high content of Cr. From the above analyses, it can be seen that the 24% Cr–76% Ni coating had most debris, but the surface was not significantly damaged. Compared to the wear morphologies and wear rates of the Cr–Ni coatings with the different mass ratios of Cr and Ni, the wear resistance of 24% Cr–76% Ni coating was the best, indicating that the increasing of Cr content improved its wear resistance.

## 4. Conclusions

(1) The laser-cladded Cr–Ni coatings with different ratios of Cr and Ni are composed of Cr, Ni and their compound phases, with no obvious defects, which are metallurgically combined with the substrate well.

(2) The average COF of 17% Cr–83% Ni, 20% Cr–80% Ni and 24% Cr–76% Ni coatings are 1.10, 0.33, and 0.87, respectively; among them, the average COF of 20% Cr–80% Ni coating is the smallest, showing higher anti–friction performance.

(3) The wear rate of 17% Cr–83% Ni, 20% Cr–80% Ni and 24% Cr–76% Ni coatings are 4.533 × 10^−6^, 5.433 × 10^−6^, and 1.761 × 10^−6^ N^−1^·s^−1^, respectively; among them, the wear rate of 24% Cr–76% Ni coating is 2–3 times smaller than that of 17% Cr–83% Ni and 20% Cr–80% Ni coatings, showing better wear resistance.

(4) The wear mechanism of 17% Cr–83% Ni and 20% Cr–80% Ni coatings at 600 °C is primarily adhesive wear, while that of 24% Cr–76% Ni coating is adhesive wear accompanied by oxidative wear. The wear performance of 24% Cr–76% Ni coating is better than those of 17% Cr–83% Ni and 20% Cr–80% Ni coatings, showing that the increase of Cr content is the main ingredient of improving the wear resistance of Cr–Ni coatings.

## Figures and Tables

**Table 1:** Chemical composition of H13 hot work mould steel/wt %.

C	Si	Mn	Cr	Mo	V	P	S	Fe
0.32–0.45	0.80–1.20	0.20–0.50	4.75–5.50	1.10–1.75	0.80–1.20	≤0.03	≤0.03	other

**Table 2:** Surface roughness of Cr–Ni coatings with different mass ratios of Cr and Ni.

Coating Kinds	Average Roughness *S*_a_/nm	Root mean Square *S*_q_/nm	Surface Skewness *S*_sk_	Surface kurtosis *S*_ku_	Ten Point Height *S*_z_/nm
17% Cr–83% Ni	3.96	6.82	−2.18	24.80	126
20% Cr–80% Ni	4.97	9.55	3.62	34.30	166
24% Cr–76% Ni	4.07	7.44	−3.35	32.1	131
